# Venous thromboembolism in COVID-19 patients and prediction model: a multicenter cohort study

**DOI:** 10.1186/s12879-022-07421-3

**Published:** 2022-05-13

**Authors:** Yi Lee, Qasim Jehangir, Pin Li, Deepthi Gudimella, Pooja Mahale, Chun-Hui Lin, Dinesh R. Apala, Geetha Krishnamoorthy, Abdul R. Halabi, Kiritkumar Patel, Laila Poisson, Venugopal Balijepally, Anupam A. Sule, Girish B. Nair

**Affiliations:** 1 Department of Medicine, St. Joseph Mercy Oakland Hospital, 44405 Woodward Avenue, Pontiac, MI 48341 USA; 2 Department of Public Health Sciences, Henry Ford Hospital, Detroit, MI USA; 3 School of Business Administration, Oakland University, Rochester, MI USA; 4 Division of Cardiology, St. Joseph Mercy Oakland Hospital, Pontiac, MI USA; 5 Oakland University William Beaumont School of Medicine, Auburn Hills, MI USA; 6 Department of Informatics, St. Joseph Mercy Oakland Hospital, Pontiac, MI USA; 7 Division of Pulmonary and Critical Care Medicine, Beaumont Hospital, Royal Oak, MI USA

**Keywords:** COVID-19, Venous thromboembolism, Deep vein thrombosis, Pulmonary embolism, Risk stratification, Risk prediction, Anticoagulation

## Abstract

**Background:**

Patients with COVID-19 infection are commonly reported to have an increased risk of venous thrombosis. The choice of anti-thrombotic agents and doses are currently being studied in randomized controlled trials and retrospective studies. There exists a need for individualized risk stratification of venous thromboembolism (VTE) to assist clinicians in decision-making on anticoagulation. We sought to identify the risk factors of VTE in COVID-19 patients, which could help physicians in the prevention, early identification, and management of VTE in hospitalized COVID-19 patients and improve clinical outcomes in these patients.

**Method:**

This is a multicenter, retrospective database of four main health systems in Southeast Michigan, United States. We compiled comprehensive data for adult COVID-19 patients who were admitted between 1st March 2020 and 31st December 2020. Four models, including the random forest, multiple logistic regression, multilinear regression, and decision trees, were built on the primary outcome of in-hospital acute deep vein thrombosis (DVT) and pulmonary embolism (PE) and tested for performance. The study also reported hospital length of stay (LOS) and intensive care unit (ICU) LOS in the VTE and the non-VTE patients. Four models were assessed using the area under the receiver operating characteristic curve and confusion matrix.

**Results:**

The cohort included 3531 admissions, 3526 had discharge diagnoses, and 6.68% of patients developed acute VTE (N = 236). VTE group had a longer hospital and ICU LOS than the non-VTE group (hospital LOS 12.2 days vs. 8.8 days, p < 0.001; ICU LOS 3.8 days vs. 1.9 days, p < 0.001). 9.8% of patients in the VTE group required more advanced oxygen support, compared to 2.7% of patients in the non-VTE group (p < 0.001). Among all four models, the random forest model had the best performance. The model suggested that blood pressure, electrolytes, renal function, hepatic enzymes, and inflammatory markers were predictors for in-hospital VTE in COVID-19 patients.

**Conclusions:**

Patients with COVID-19 have a high risk for VTE, and patients who developed VTE had a prolonged hospital and ICU stay. This random forest prediction model for VTE in COVID-19 patients identifies predictors which could aid physicians in making a clinical judgment on empirical dosages of anticoagulation.

**Supplementary Information:**

The online version contains supplementary material available at 10.1186/s12879-022-07421-3.

## Introduction

Severe Acute Respiratory Syndrome Coronavirus 2 (SARS-CoV-2) has been causing COVID-19 illness globally since December 2019, with more than 310 million people infected and more than five million deaths reported as of 1st Jan 2022 [1]. The common manifestations of COVID-19 include fever, cough, dyspnea, myalgia, fatigue, and diarrhea. Primarily, COVID-19 infection results in respiratory complications. However, it is evident that COVID-19 infection may be associated with a hyper-coagulable state, which leads to microvascular and macrovascular arterial and venous thromboembolism (VTE) [2, 3].

The incidence of VTE complications in COVID-19 patients ranged from 1.7 to 16.5% in 35 observational studies reported from around the world (total N = 9249) [4]. Researchers postulated that a severely activated inflammatory response to COVID-19 infection causes thrombo-inflammation; through mechanisms such as cytokine storm, complement activation, and endotheliosis [5]. In addition, certain studies reported findings of microthrombi in autopsies of COVID-19 patients [6]. Recent retrospective studies proposed several risk factors associated with higher mortality and higher severity of COVID-19, including inflammatory markers such as interleukin-6 (IL-6), D-dimer, ferritin, and lactate dehydrogenase (LDH)[7, 8]. Moreover, many studies also showed VTE in COVID-19 is associated with severity of infection and mortality [8]. Hence it is critical for physicians to identify the risk factors for the prevention and early management of VTE.

Most of the prediction models built for COVID-19 patients predict prognosis [9–11], with only a few models predicting VTE [12–14]. These models were built using a limited selection of variables, mostly had a smaller sample size, and primarily involved modification and validation of pre-COVID-19 VTE prediction models. With the growing awareness of VTE risk in COVID-19, patients are now routinely placed on prophylactic dose anticoagulants per National Institute Health recommendation, except in cases of high bleeding risk, severe thrombocytopenia, or suspected hemorrhage necessitating caution in these selected patients [6, 15–17]. This highlights the need for a prediction model tailored for COVID-19 patients, with comprehensive variable selection and performance evaluation, which can support the use of anticoagulation in this crucial patient population. Therefore, we analyzed the independent predictors of VTE using different machine learning methods in a cohort of 3531 hospitalized COVID-19 patients from Southeastern Michigan.

## Methods

In this cross-sectional retrospective observational study, we report and analyze the data from Southeastern Michigan COVID-19 Consortium Registry Database (SMCRD). As previously described, SMCRD is a multi-institutional registry database of four main health systems in Southeast Michigan, United States, including Henry Ford Health System, Beaumont Health System, Trinity Health System, and Wayne State University [18]. It is built using REDCap and is housed at Vanderbilt University Medical Center. The SMCRD registry contains de-identified data of adult patients who were hospitalized with laboratory-confirmed SARS-CoV-2 PCR tests. Each institution independently collected data from March 1, 2020, to September 5, 2021. Our study was approved by the institutional review board (IRB) of Trinity Health System.

### Procedures

**Fig. 1:**
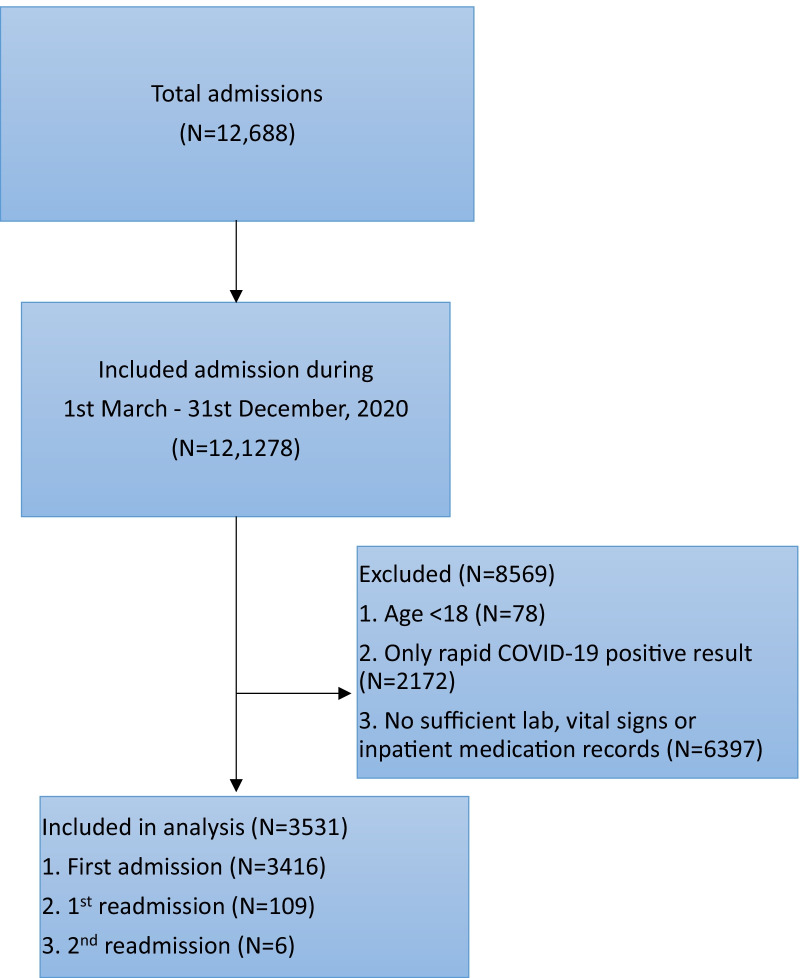
Consort diagram of Southeastern Michigan COVID-19 Registry Consortium Database

We compiled data for adult patients (age 18 years or older) that included baseline demographics, laboratory results, and in-hospital events, including all-cause mortality of COVID-19 patients from March 1, 2020, to the end of December 2020. All patients (with and without VTE events) were included (Fig. 1). For each patient, a total of 85 variables (Additional file 1: Table S1) from six categories were extracted, including baseline demographics, presenting vital signs, past medical history (abstracted using standard-text variables, International Classification of Diseases–Tenth Revision (ICD-10) and Current Procedural Terminology codes), social history, admission reasons, pre-admission and in-hospital medications, hospital course, laboratory values, electrocardiogram, and imaging studies (magnetic resonance imaging (MRI), computerized tomography scan, ultrasounds). Variables in our study included: personal information (age, sex, race/ethnicity, body mass index (BMI), social history), hospital summary (hospital length of stay (LOS), intensive care unit (ICU) admission and LOS, use of oxygen devices, intubation status), laboratory values (white blood cell (WBC) counts, D-dimer, ferritin, LDH, lactate, C-reactive protein (CRP), and so on), past medical history, vital signs, and in-hospital prophylactic and therapeutic anticoagulation therapy. Since COVID-19 can cause VTE in patients following discharge, we followed patients after their initial hospital discharge for readmission and development of VTE. Accordingly, patients with one-time admission and readmissions, with or without thromboembolism events, were considered when building prediction models.

### Outcomes

The primary outcome was in-hospital VTE events, including acute deep vein thrombosis (DVT) and pulmonary embolism (PE) identified by ICD-10 codes (Additional file 1: Table S2), venous Doppler ultrasounds, ventilation-perfusion scan, and computed tomography angiography (CTA) of the chest. In-hospital outcomes (Table 1) included mortality, and hospital and ICU LOS.

**Table 1:** Baseline characteristics of COVID-19 patients with and without acute venous thromboembolism

Variable			No VTE	Acute VTE	*p* value
Gender		Male	1447 (49.8)	118 (50.0)	1
		Female	1460 (50.2)	118 (50.0)	
Race/ethnicity		American Indian or Alaskan Native	6 (0.2)	0 (0.0)	0.119
		Asian or Pacific Islander	52 (1.8)	0 (0.0)	
		Black	995 (34.2)	93 (39.4)	
		White	1645 (56.6)	131 (55.5)	
		Hispanic	50 (1.7)	1 (0.4)	
		Others	86 (3.0)	4 (1.7)	
		Unknown	72 (2.5)	7 (3.0)	
Age (years)		Mean (SD)	66.2 (16.4)	68.0 (16.7)	0.125
Body mass index (kg/m^2^)		< 18.5	52 (1.9)	7 (3.3)	0.329
		18.5–24.9	487 (17.7)	44 (20.8)	
		25–29	828 (30.2)	60 (28.3)	
		> 30	1378 (50.2)	101 (47.6)	
Hospital LOS (days)		Mean (SD)	8.8 (6.4)	12.2 (9.2)	< 0.001
Total ICU (days)		Mean (SD)	1.9 (5.0)	3.8 (8.1)	< 0.001
Mechanical ventilation (days)		Mean (SD)	1.1 (3.9)	2.4 (6.9)	0.005
Cardiopulmonary resuscitation			420 (14.4)	37 (15.7)	0.675
Oxygen device		None	2241 (77.4)	153 (64.8)	< 0.001
		Nasal cannula/non-rebreather mask	574 (19.8)	60 (25.4)	
		Ventilator	43 (1.5)	11 (4.7)	
		Other	29 (1.0)	5 (2.1)	
		High-flow nasal cannula	7 (0.2)	7 (3.0)	
Vitals on presentation	Oxygen saturation (%)	Mean (SD)	94.0 (6.2)	93.4 (7.2)	0.258
	Heart rate (beats/minute)		94.3 (19.7)	100.7 (20.7)	< 0.001
	Respiratory rate (breaths/minute)		20.9 (6.4)	21.3 (6.0)	0.283
	Diastolic blood pressure (mmHg)		74.7 (15.6)	74.6 (16.5)	0.897
	Systolic blood pressure (mmHg)		134.1 (24.9)	130.9 (25.6)	0.074
Labs on presentation	White blood cell count (K/uL)		7.5 (4.5)	9.4 (6.0)	< 0.001
	Lymphocytes (K/uL)		1.6 (1)	1.4 (0.9)	0.007
	Neutrophils (K/uL)		6.7 (2.5)	6.6 (2.8)	0.645
	Hemoglobin A1c (%)		7.8 (2.2)	7.4 (2.2)	0.281
	B-type natriuretic peptide (pg/mL)		167.2 (338.2)	189.5 (311.9)	0.387
	C-reactive protein (mg/dL)		9.2 (7.6)	11.6 (8.5)	< 0.001
	D-dimer (μg/mL)		1.8 (2.3)	4.6 (5.0)	< 0.001
	Ferritin (ng/mL)		753.3 (1673.9)	725.7 (978.0)	0.716
	Fibrinogen (mg/dL)		570.5 (168.3)	554.0 (207.1)	0.573
	Interleukin-6 (pg/mL)		76.3 (127.4)	136.5 (159.8)	0.094
	)Hemoglobin (gm/dL)		10.9 (2.9)	12.4 (5.1)	0.754
	Lactate (mmol/L)		1.8 (1.4)	2.0 (1.3)	0.384
	Lactate dehydrogenase (U/L)		322.9 (340.8)	370.1 (367.3)	0.079
	Alanine transaminase (U/L)		53.1 (205.8)	64.5 (204.0)	0.436
	Aspartate aminotransferase (U/L)		59.9 (216.8)	100.3 (564.9)	0.298
	Procalcitonin (ng/mL)		2.1 (8.7)	1.3 (2.2)	0.008
	Blood urea nitrogen (mg/dL)		26.0 (21.3)	30.3 (26.0)	0.018
	Creatinine (mg/dL)		1.6 (2.0)	1.6 (1.9)	0.711
	Potassium (meq/L)		4.0 (0.7)	4.1 (0.8)	0.034
	Total bilirubin (mg/dL)		0.8 (0.6)	1.0 (2.6)	0.227
	Platelet count (K/uL)		218.1 (95.3)	258.7 (144.2)	< 0.001
Social history	Smoker		216 (7.4)	19 (8.1)	0.826
	Alcohol Use		45 (1.5)	7 (3.0)	0.168
	Marijuana Use		18 (0.6)	3 (1.3)	0.443
In-hospital medications	Inpatient anticoagulation therapeutic dose		302 (11.5)	101 (46.3)	< 0.001
	Inpatient anticoagulation prophylactic dose		2446 (92.8)	162 (74.3)	< 0.001
Home medications	Non-steroidal anti-inflammatory drugs		29 (10.9)	5 (18.5)	0.384
	Azithromycin		21 (7.9)	4 (14.8)	0.383
	Hydroxychloroquine		4 (1.5)	1 (3.7)	0.949
	Angiotensin-converting enzyme inhibitors		66 (24.7)	9 (33.3)	0.455
	Angiotensin—ll receptor blockers		42 (15.7)	5 (18.5)	0.919
	Beta blockers		91 (34.1)	7 (25.9)	0.52
	Diuretics		85 (31.8)	9 (33.3)	1
	Statins		115 (43.1)	8 (29.6)	0.252
	Warfarin		12 (4.5)	2 (7.4)	0.839
	Aspirin		88 (33.0)	5 (18.5)	0.187
	P2Y12 inhibitors		10 (3.7)	2 (7.4)	0.685
	Direct-acting oral anticoagulants		17 (6.4)	0 (0.0)	0.359
	Other anticoagulants		7 (2.6)	0 (0.0)	0.85
	Corticosteroids		26 (9.7)	4 (14.8)	0.619
	Proton pump inhibitors		70 (26.2)	10 (37.0)	0.329
Oxygen requirement prior to admission	Yes		18 (6.7)	0 (0.0)	0.319
	Unknown		5 (1.9)	1 (3.7)	
Lab values (Maximum and Minimum)	White blood cell count Max	Mean (SD)	8.8 (5.3)	10.4 (6.2)	< 0.001
	Lymphocytes Min		12.3 (7.6)	11.0 (7.5)	0.013
	Neutrophils Max		84.0 (10.6)	86.0 (10.5)	0.007
	Hemoglobin A1c Min		7.8 (2.2)	7.4 (2.2)	0.291
	B-type natriuretic peptide Max		168.8 (333.9)	197.0 (318.8)	0.283
	C-reactive protein Max		7.6 (5.8)	9.8 (8.1)	< 0.001
	D-dimer Max		2.3 (2.5)	4.7 (3.8)	< 0.001
	Ferritin Max		871.4 (2476.0)	773.6 (1094.9)	0.284
	Fibrinogen Max		594.3 (168.9)	571.8 (196.6)	0.423
	Interleukin-6 Max		79.3 (137.6)	138.3 (160.5)	0.104
	Hemoglobin Min		10.0 (2.4)	10.6 (0.5)	0.445
	Lactate Max		2.0 (1.5)	2.4 (2.7)	0.165
	Lactate dehydrogenase Max		380.0 (369.0)	438.1 (378.4)	0.036

*ICU* intensive care unit, *Max* maximum, *Min* minimum, *LOS* length of stay, *SD* standard deviation, *VTE* venous thromboembolism

### Statistical analysis

#### Initial data cleaning and analysis

Laboratory values at the time of admission, peak, and minimum values were collected. For VTE, approximately 5% of patients had CTA chest images available, and 1% of patients had CTA-confirmed PE and vessel image-confirmed DVT; limited diagnostic testing was likely due to the COVID-19 hospitals’ policy of limiting exposure to the virus in the first wave of the pandemic. Of the 3531 patients, 161 patients had PE, and 121 had DVT. 3127 patients were anticoagulated with either enoxaparin or heparin. Enoxaparin dosage higher than 40 mg subcutaneous twice daily was considered as therapeutic dose (N = 340), whereas less than 40 mg subcutaneous twice daily was defined as prophylactic dose (N = 1920). Intravenous heparin was included in the therapeutic dose (N = 182) and subcutaneous heparin was considered as the prophylactic dose (N = 1315). In total, 1018 patients received therapeutic dose and 2976 patients received prophylactic dose anticoagulation.

We categorized race/ethnicity, BMI, oxygen devices, smoking, alcohol and marijuana history, and past medical history into dichotomous variables, while laboratory test values were retained as continuous variables. Initial descriptive analysis for continuous variables was described as mean with standard deviation or median with interquartile range. Categorical variables were described as frequency distributions. To compare the groups, the Chi-square test was used for categorical variables, and the t-test was used for continuous variables. Univariate analysis and principal component analysis (PCA) were used to identify potential risk factors for VTE (Additional file 1: Table S3 and Fig. S1). All data were analyzed using SAS v9.4 or R 3.6.2, and a p-value less than 0.05 was considered to indicate statistical significance. Prediction models were built using JMP Pro 14.2.0 (Additional file 1: Table S4).

### Data cleaning

As part of exploratory data analysis, the distribution of all the variables was plotted. Most laboratory values were either left or right-skewed. Multiple variables could be highly correlated with each other and potentially result in interactions in the process of model building. For example, both neutrophil and lymphocyte counts comprise the neutrophil–lymphocyte ratio. Likewise, BUN and creatinine comprise the BUN-creatinine ratio, which is a parameter that could indicate different types of acute kidney injury; for example, the BUN-creatinine ratio > 20 suggests pre-renal acute kidney injury. Therefore, Spearman’s rho was performed. Twenty-three groups of variables that were highly positively or negatively correlated based on Spearman’s coefficient of more than ± 0.7 (Additional file 1: Table S5A) were aspartate aminotransferase (AST) and alanine transaminase (ALT), creatinine and BUN, maximum (max) B-type natriuretic peptide (BNP) and initial BNP, max CRP and initial CRP, max ferritin and initial ferritin, max D-dimer and initial D-dimer, neutrophil–lymphocyte ratio and neutrophils, max neutrophils and minimum lymphocyte, history of VTE, DVT and PE, systolic blood pressure and diastolic blood pressure, inpatient therapeutic anticoagulation and inpatient prophylactic anticoagulation and so on. Therefore, we downsized the variables; for example, neutrophil and lymphocyte alone were analyzed in the model building rather than the neutrophil–lymphocyte ratio. Likewise, BUN and creatinine alone were included rather than the BUN-creatinine ratio; the history of VTE was used rather than its components (DVT and PE) (Additional file 1: Table S5B). When building models, we used lab values on admission rather than the peak or lowest values as we aimed to build a prediction model which can assist physicians in predicting VTE in COVID-19 patients on admission based on the available data. The PCA was performed to reduce the dimensions used to predict VTE events. Patients without missing data (N = 1443) from the cohort were included in the PCA. A total of 32 continuous variables were included in the PCA. In the scree plot, the 1st component explained only about 16% of variations of the data, and only 24.6% of the variations were explained by the first two components (Additional file 1: Table S3 and Fig. S1). Therefore, the PCA was deemed not helpful in reducing the dimensions in our analysis. For both continuous and categorical variables, we further performed univariate analysis using the R packages (Additional file 1: Table S4).

### Model building

The cohort was randomly split into the training set and test set (70:30) multiple times. We compared four models in their predictive accuracy for detecting VTE events and mortality:

- Multiple linear regression (MLR)
- Multiple logistic regression (LR)
- Decision tree
- Random forest

## Results

A total of 3531 admissions were identified, of which 3416 were first admissions and 115 were readmissions; of the 115 readmitted patients, 109 were readmitted once, and 6 were readmitted twice. Overall, there were 236 patients (6.68%) with VTE events and 2907 patients with no VTE events in the dataset. In general, the VTE group had a longer LOS in hospital and ICU than the non-VTE group (hospital LOS 12.2 days vs. 8.8 days, p < 0.001; ICU LOS 3.8 days vs. 1.9 days, p < 0.001). In addition, 9.8% of patients in the VTE group required advanced oxygen support, compared to 2.7% of patients in the non-VTE group (p < 0.001). Laboratory values such as WBC, CRP, D-dimer, and platelet count were significantly different between VTE and non-VTE groups (p < 0.001). Baseline demographic characteristics of patients are summarized in Table 1. The mean age for VTE and non-VTE patients was 68 ± 16.7 years and 66.2 ± 16.4 years (p = 0.125), respectively. Morbid obesity was common in both groups (VTE vs. non-VTE: 47.6% vs. 50.2%, p = 0.329). The in-hospital all-cause mortality for VTE patients was 22.2%, whereas non-VTE patients was 14.8% [Odds ratio (OR): 1.65, 95% confidence interval (CI): 1.22, 2.22, p = 0.001]. We also found that the VTE group had a longer hospital LOS, ICU LOS, and days on ventilator than the non-VTE group. The univariate analysis of predictors of VTE upon admission are shown in Additional file 1: Table S3. The variables like IL-6 (pg/mL), CRP (mg/dL), D-dimer (ng/mL), WBC (K/uL), BUN (mg/dL) had an OR of 1.00 to 1.2 and were significant; this was not negligible as most of the variables were measured on a small scale. Moreover, these laboratory variables are of great interest in COVID-19 patients because COVID-19 infection causes cytokine storm leading to elevated inflammatory markers, such as ferritin, LDH, CRP, and IL-6. These inflammatory responses result in endotheliitis and hypercoagulopathy that predispose the patients to develop VTE.

### Prediction model for VTE

The most significant variables of each model are shown in Table 2. For MLR and LR, the significant variables were selected based on the p-value of < 0.05; for decision tree and random forest, they were based on the Gini index. MLR was eliminated as it is not ideal for categorical variables. The decision tree has worse accuracy than a random forest but provides interpretability. Our decision tree was firstly split by the root node as therapeutic anticoagulation as inpatient, followed by leaf nodes of BUN (< 20, 20), hospital LOS (< 20, 20), Age (< 91, 91), race (White, non-White), D-dimer (4740 ng/mL, < 4740 ng/mL), history of VTE, and D-dimer (2170 ng/mL, < 2170 ng/mL) (Additional file 1: Fig. S2). Whereas random forests are an ensemble of decision trees that solve the overfitting of the decision tree as the predictions are based on an average of all trees. On the other hand, loss of interpretability is one of the limitations of the random forests. Both decision trees and random forests handle continuous and categorical variables that best analyze our cohort. Across all models, D-dimer was the most significant variable for MLR, LR, and decision tree models. Other common variables across the models include VTE history, inpatient therapeutic anticoagulation, requirement for oxygen devices such as high-flow nasal cannula, non-rebreather mask, and mechanical ventilation, heart rate, BUN, and so on. The four models were compared, as shown in Table 3, to analyze predictive ability in diagnosing COVID-19 associated VTE. Random forest performed the best among all in terms of R-square (R^2^), misclassification rate, and receiver operating characteristic (ROC) curve.

**Table 2:** Significant variables in prediction models, listed in descending order: (1) Multiple linear regression (2) Multiple logistic regression (3) Decision tree (4) Random forest

Multiple linear regression	Multiple logistic regression	Decision tree	Random forest
D-dimer	D-dimer	Therapeutic anticoagulation inpatient	D-dimer
Nonalcoholic steatohepatitis	History of venous thromboembolism	History of venous thromboembolism	Therapeutic anticoagulation inpatient
History of venous thromboembolism	Mechanical ventilation	D-dimer	Platelet count
Therapeutic anticoagulation inpatient	Therapeutic anticoagulation inpatient	Age	Blood urea nitrogen
High-flow nasal Cannula	High-flow nasal Cannula	Race/ethnicity	Age
Mechanical ventilation	Nonalcoholic steatohepatitis	Blood urea nitrogen	WBC count
Coronary artery bypass graft	Nonalcoholic steatohepatitis	Hospital length of stay	Systolic blood pressure on presentation
Heart rate on presentation	Thyroid disease		Lymphocytes
Alanine aminotransferase	Nasal Cannula or non-rebreather Mask		Alanine aminotransferase
Chronic kidney disease	Coronary artery bypass graft		Abnormal potassium level (higher or lower)
	Chronic kidney disease		B-type natriuretic peptide
	Ferritin		C-reactive protein
			Creatinine
			Lactate dehydrogenase
			Neutrophils
			Heart rate on presentation
			Total bilirubin
			Aspartate transaminase
			Diastolic blood pressure on presentation
			Venous thromboembolism
			Ferritin
			Oxygen saturation on presentation

**Table 3:** Model performance for venous thromboembolism prediction in COVID-19 patients

Model	Misclassification Rate		R-square		AIC	BIC	Sensitivity	Specificity	PPV	NPV	AUC
	TS	VS	TS	VS							
Multiple linear regression	NA	NA	25.39%	16.29%	50	355	NA	NA	NA	NA	NA
Multiple logistic regression	5.74%	9.64%	41.12%	3.79%	436	742	0.76	0.76	0.87	0.85	0.80
Decision tree	7.11%	9.65%	19.89%	11.35%	NA	NA	0.69	0.65	0.78	0.79	0.77
Random forest	6.84%	8.40%	58.89%	18.76%	NA	NA	0.68	0.82	0.26	0.97	0.83

*AIC* Akaike information criterion, *AUC* area under the curve, *BIC* Bayesian information criterion, *NA* not applicable, *NPV* negative predictive value, *PPV* positive predictive value, *TS* training set, *VS* validation set

### Performance of the model

**Fig. 2:**
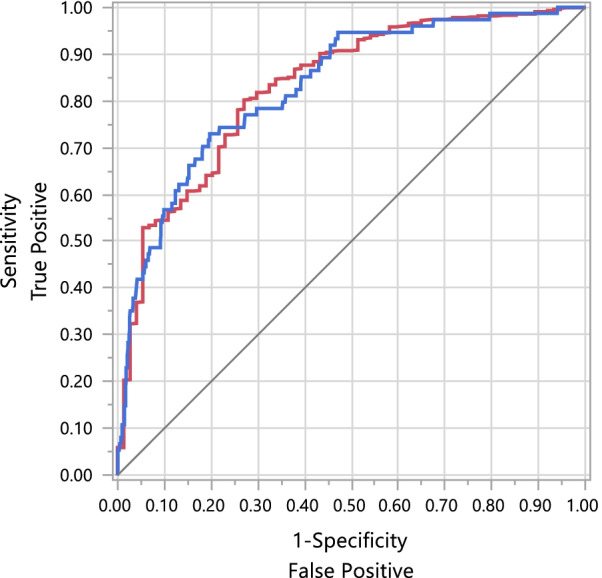
Receiver operating characteristic (ROC) curve of the random forest model for venous thromboembolism in COVID-19 patients. The random forest model’s area under the ROC curve was 0.83

Random forest model consisted of 22 variables (significance in order): D-dimer, inpatient therapeutic anticoagulation therapy, platelet count, BUN, age, WBC, systolic blood pressure, lymphocytes, ALT, potassium, BNP, CRP, creatinine, LDH, neutrophils, heart rate, total bilirubin, AST, diastolic blood pressure, prior history of VTE, ferritin, and oxygen saturation on admission. Electrolytes, renal function, blood pressures, hepatic enzymes, and inflammatory markers were indicators of VTE risks. The evaluation of the performance and confusion matrix of the four models in training and the validation process is shown in Table 3. The R^2^ of the random forest model for the training and validation set was 58.87% and 18.76% (p < 0.0001); the area under the ROC curve was 0.83 (Fig. 2). We set a cutoff of 0.1 for the generation of sensitivity and specificity. The random forest model had a sensitivity of 0.68 and a specificity of 0.82. In our cohort, the classification was skewed; therefore, the default threshold (0.5) cannot represent an optimal interpretation of the predicted probabilities. Effectively, our goal was to provide a robust model for clinicians to identify COVID-19 patients at risk for VTE early in the hospital course and assist in deciding between therapeutic versus prophylactic anticoagulation management. In the validation set, the model showed that it was good at predicting the absence of a venous event more than the presence of a venous event. The negative predictive value (NPV) and positive predictive value (PPV) of the model for the validation set were 0.97 and 0.26. Due to the low prevalence of VTE in the population, the F1 score of the model was calculated as 0.35.

## Discussion

VTE is one of the most common complications in COVID-19 patients [19–22]. This retrospective study presents a prediction model for VTE in COVID-19 patients and the demographics, clinical parameters, and incidence rate of VTE in COVID inpatients. The incidence rate of VTE could have been underreported due to limited radiological testing to reduce staff exposure to COVID-19 infection in the first wave [23]. Our study reported an incidence rate of 6.68%, similar to other studies [24–29] (Table 4). We found that patients who developed new-onset VTE had more extended hospital LOS (12.2 days vs. 8.8 days, p < 0.001) and ICU LOS (3.8 days vs. 1.9 days, p < 0.001) compared to patients who did not have VTE. This is a robust prediction model for VTE in hospitalized patients with COVID-19 using a large multicenter database (N = 3531). We included 85 variables from a broad spectrum of parameters, demographics, vitals, comorbidities, and hospital course (oxygen requirement, ICU admission, hospital and ICU LOS). Electrolytes, renal function, blood pressures, hepatic enzymes, and inflammatory markers were indicators of VTE risks; however, further studies on whether a cutoff value could be applied to inflammatory markers for good sensitivity and specificity for VTE in COVID-19 infection would be beneficial. Physicians can assess patients’ presenting signs, renal and hepatic functions, and potentially identify patients at high risk of VTE and work on the reversible risk factors to reduce patients’ risks of developing VTE during hospitalization.

**Table 4:** Characteristics of retrospective COVID-19 studies on venous thromboembolism incidence rate and predictors

Study	Country	Study type, time period	Total number of cases	Venous thromboembolism incidence rate	Analysis performed	Identified predictors
Cohen et al.	United States	Retrospective, March 1st to April 27th, 2020	9407	2.9% (2.4% in medical ward and 4.9% in ICU)	Multivariate analysis	For VTE or mortality: 1.Advanced age 2.Increasing Charlson Comorbidity Index 3.History of cardiovascular disease 4.ICU level of care, and 5.Elevated maximum D-dimer with a cutoff at least four times the upper limit of normal
Dalager- Pedersen et al.	Denmark	Retrospective, January 27th to June 1st, 2020	1540	5% VTE (both ICU and general ward)	30-day absolute risks	This Study compared COVID-19 and non-COVID-19 patients showed COVID-19 patients had a higher risk of VTE
Freund et al.	France, Spain, Belgium, Italy, Chile, Canada	Retrospective, February 1st to April 10th, 2020	974	15% (only PE, DVT not studied)	Multivariable binary logistic regression	1. Male gender 2. Age > 48 3. Heart rate 4. Prior history of VTEs 5. Clinical signs of DVT 6. Recent immobilization
Mei et al.	China	Retrospective, January 1st to March ﻿23rd, 2020	616	2% VTE (DVT and/or PE)	χ^2^ test, Fisher exact test, t test, and Mann–Whitney U test	This study compared Padua score in COVID-19 pneumonia and community-acquired pneumonia
Poissy et al.	France	Retrospective case series, February ﻿27th to March 31st, 2020	196 (ICU patients only)	6.1% (PE only)	Simple descriptive analysis	None
Rieder et al.	China	Retrospective, March 26th to April 20th, 2020	49	6.1%	Spearman test	The level of D-dimers at hospital admission and the maximum level during follow-up were correlated with days at the hospital, days in ICU, days on non-invasive ventilation, or days on invasive ventilation

*DVT* deep vein thrombosis, *ICU* intensive care unit, *PE* pulmonary embolism, *VTE* venous thromboembolism

**Table 5:** Characteristics of retrospective studies on venous thromboembolism prediction models

Study	Country	Study type, time period	Total number of cases	Venous thromboembolism incidence rate	Prediction model	Performance
Kampouri et al.	Switzerland	Retrospective, February 28th to April 30th, 2020	491	9.3%	Wells score for PE ≥ 2 points and D-dimer value ≥ 3,000 ng/mL	PPV: 18.2% NPV: 98.5 Accuracy: 0.905
Dujardin et al.	Netherlands	Retrospective, March 13th to April 9th, 2020	127	41.7%	Binary linear regression model; D-dimer is > 9 μg/mL and C-reactive protein > 280 mg/mL	Predicted probability: 92%
Tsaplin et al.	Russia	Retrospective, April 30th to May 29th, 2020	168	6.5%	Modified Caprini score > 12; D-dimer > 3 upper limit of normal	Sensitivity: 73%; Specificity: 84%
Spyropoulos et al.	United States	Retrospective, March 1st, 2020 to April 27th, 2020	9407	2.9%	The International Medical Prevention Registry on Venous Thromboembolism and D‐Dimer (IMPROVE‐DD) risk assessment model	AUC: 70%; sensitivity: 97%; specificity: 22%
Freund et al.	France, Spain, Belgium, Italy, Chile, and Canada	Retrospective, February 1st to April 10th, 2020	974	15%	Revised Geneva score and D-dimer [D-dimer below the age-adjusted threshold (i.e., 500 µg/mL under 50 years and age × 10 over 50 years)]	AUC: 0.81

*AUC* area under the curve, *NPV* negative predictive value, *PE* pulmonary embolism, *PPV* positive predictive value

It is worth mentioning that we used presenting data which was the initial data of patients admitted to the hospital. Models such as multiple LR models that do not handle missing data have smaller sample sizes that can potentially affect performance. Our MLR model has an R^2^ of 0.2569, p < 0.0001. The R^2^ value of MLR and LR is low, which is consistent with the fact that we did not include laboratory values that are missing and did not impute those values. The decision tree has a lower R^2^ value (0.19 in training and 0.11 in the testing set). However, the R^2^ value is most likely not appropriate for a tree-based model. Nevertheless, the random forest model has a low misclassification rate (6.87% in the training set, 8.4% in the testing set). Overall, we have low R^2^ values. The decision tree may have worse accuracy than a random forest, but the tree structure is easy to understand and interpret. By looking at the splitting nodes, key factors can be identified, and predictions can be made. On the other hand, random forests are an ensemble of decision trees, and the predictions are based on an average of all trees, which is a “black box” that can’t be directly described. One of the possibilities is that our study cohort has an inherently higher amount of unexplainable variability; this could be better addressed in future prospective studies.

Of 3532 records, only 1282 patients were included in the MLR model due to the missing values in the other patients. Similarly, in the LR, only 1282 records were used, which was less than 50% of the records. Although IL-6, LDH, procalcitonin, ferritin, and fibrinogen were excluded in the model building due to significant numbers of missing values, we found no significant difference in these values between non-VTE and VTE groups.

Our model can provide clinical risk stratification of VTE in COVID-19 patients and help individualize thromboprophylaxis, which supports the current consensus of customized and risk-adapted management for thromboprophylaxis in international guidelines [30]. Five papers studied VTE in COVID-19 patients using existing prediction models [26, 31–34] (Table 5). Kampouri et al. combined the Wells score and D-dimer value to predict VTE with a PPV of 18.2%, an NPV 98.5%, and accuracy of 0.905 [31]. A Dutch study reported a 41.7% incidence rate of VTE in COVID-19 patients and built a linear regression model consisting of D-dimer > 9 μg/mL and CRP > 280 mg/mL, and the authors report a predicted probability of 92% [32]. Another study by Taplin et al. modified the Caprini score using a cutoff value of 12, which is also based on the D-dimer score and showed a sensitivity of 73% and specificity of 84% in predicting VTE [33]. Unlike our study, most of these studies had a smaller sample size and number of events and included risk factors not analyzed in the original prediction model studies. Notably, the performance of the model depends on the event prevalence. Among all studies, the Dutch study had the highest predictive probability in the critically ill population due to a higher incidence of VTE [32]. A meta-analysis of 47 studies showed high prevalence of PE with high mean D-dimer values (prevalence ratio 1.3 per 1000 ng/mL increase; 95% CI: 1.11, 1.50, p = 0.002) and percentage of ICU patients (1.02 per 1% increase; 95% CI: 1.01, 1.03, p < 0.001). In addition, prevalence of DVT was also high across studies with high mean D-dimer values (1.04 per 1000 ng/mL increase; 95% CI: 1.01, 1.07, p = 0.022)[35].

After systemic review, we included six studies that reported VTE incidence rate in COVID-19 patients without prediction models (Table 4). Our study showed an incidence rate of 6.68% of VTE in COVID-19 patients, which is consistent with three of the studies [25, 28, 29], whereas Freund et al. reported a rate of 15% and two studies showed a lower incidence rate of 2–3% [24, 26, 27]. Critically ill COVID-19 patients who were admitted to ICU had a higher incidence rate of VTE. Only two studies identified risk factors for COVID-19 patients using the MLR model, including advanced age, increased Charlson Comorbidity Index, history of cardiovascular disease, ICU admission, elevated D-dimer, male gender, heart rate, clinical signs of DVT, and recent immobilization [24, 26]. Unlike other studies, we did not impute missing values to better build a model that predicts VTE individually.

Our study analyzed D-dimer, lactate, and inflammatory markers, including CRP, ferritin, and LDH that are of great interest in clinical settings and have been routinely ordered for COVID-19 patients. The utilization of laboratory values varies; many physicians trend these markers to predict the trajectory of COVID-19 patients. However, limited studies included them for VTE analysis. Our result showed no significant difference in presenting CRP, IL-6, and LDH levels among VTE and non-VTE groups (Table 1), yet the maximum value of D-dimer, CRP, and LDH were significantly higher in VTE groups. This may suggest that D-dimer, CRP and LDH could be utilized clinically for monitoring. However, further studies on the threshold, sensitivity, and specificity of certain markers are needed.

Current guidelines by the American Society of Hematology (ASH) suggest using prophylactic-intensity over intermediate-intensity anticoagulation for patients with COVID-19 related critical illness who do not have suspected or confirmed VTE [36]. Furthermore, ASH suggests that an individualized assessment of the patient’s risk of thrombosis and bleeding is important when deciding on anticoagulation intensity. Our study provides physicians with a model that could aid in risk stratification, as VTE has been well-known to be a common COVID-19 complication.

We observed that 11.5% of patients (N = 302) who did not have VTE were given a therapeutic dosage of anticoagulation, whereas 46.3% (N = 101) with VTE received therapeutic anticoagulation. It is unclear why after diagnosis of VTE, over half of the patients only received prophylactic anticoagulants. It described an unmet need for risk stratification for COVID-19 patients. Vaughn et al. reported that 16.2% of patients who had suspected VTE were given therapeutic anticoagulation and increased treatment-dose anticoagulation for VTE prophylaxis [37]. The INSPIRATION trial did not show the difference in routine empirical use of intermediate-dose prophylactic anticoagulation compared to standard dose in ICU patients with the primary composite outcome including acute VTE, arterial thrombosis, the use of extracorporeal membrane oxygenation, and all-cause mortality [absolute risk difference, 1.5% (95% CI: − 6.6, 9.8); OR: 1.06 (95% CI: 0.76, 1.48); p = 0.70] [16]. The Anti-Thrombotic Therapy to Ameliorate Complications of COVID-19 (ATTACC) randomized multicenter adaptive design trials have shown therapeutic anticoagulation to be beneficial in moderately ill patients, whereas it was futile in ICU patients requiring organ failure support [38, 39].

Our study has both strengths and limitations. The strengths include the large sample size, multi-institute-based data, and availability of broad outcomes events data. Moreover, our VTE prediction model in COVID-19 patients can most benefit clinical practice to aid clinical management in settings where a definitive diagnosis of VTE is hard to obtain, for example, for critically ill patients on mechanical ventilation who are unable to undergo CTA chest study. Since this is a retrospective study utilizing a large database, we were unable to obtain the timing of diagnosis of acute VTE in our cohort, which would have allowed exploration of the temporal relationship between VTE and potential risk factors, highlighting an important limitation of our study. Furthermore, although our models showed good predictive capacity, the lower incidence of VTE in the population study created significant hurdles. The random forest model’s PPV is 26%, NPV is 97%, and the F1 score is 0.36. Future studies on a composite outcome including both venous and arterial events could provide a bigger population. Also, the random forest model is not a panelized method and has the risk of overfitting. Lastly, our model needs to be validated externally.

## Conclusions

There is a high incidence of VTE in hospitalized COVID-19 patients. Prolonged hospital and ICU stay was noted in patients who developed VTE. This random forest prediction model for VTE in COVID-19 patients is based on a broad spectrum of parameters available on initial presentation and comorbidities. Factors like D-dimer, LDH, platelet count, age, WBC, AST, ALT, BUN and creatinine, heart rate on presentation, and prior history of VTE can predict in-hospital VTE events which could aid physicians in making a clinical judgment on the empirical dosage of anticoagulation.

## Supplementary Information

**Additional file 1.** Supplemental materials of COVID-19 venous thromboembolism prediction model.

## Data Availability

Deidentified clinical data supporting the analysis in this publication will be made available from the corresponding author upon request.

## Abbreviations

AIC: Akaike information criterion
ALT: Alanine transaminase
AST: Aspartate aminotransferase
ATTACC: Anti-Thrombotic Therapy to Ameliorate Complications of COVID-19
AUC: Area under the curve
BIC: Bayesian information criterion
BMI: Body mass index
BNP: B-type natriuretic peptide
BUN: Blood urea nitrogen
CI: Confidence interval
CRP: C-reactive protein
CTA: Computed tomography angiography
DVT: Deep vein thrombosis
ICD-10: International Classification of Diseases–Tenth Revision
ICU: Intensive care unit
IL-6: Interleukin-6
IRB: Institutional review board
LDH: Lactate dehydrogenase
LOS: Length of stay
LR: Logistic regression
Max: Maximum
Min: Minimum
MLR: Multiple linear regression
NA: Not applicable
NPV: Negative predictive value
OR: Odds ratio
PCA: Principal component analysis
PE: Pulmonary embolism
PPV: Positive predictive value
R^2^: R-square
ROC: Receiver operating characteristic
SARS-CoV-2: Severe acute respiratory syndrome coronavirus 2
SD: Standard deviation
SMC: Southeastern Michigan COVID-19 Consortium
SMCRD: Southeastern Michigan COVID-19 Consortium Registry Database
TS: Training set
VS: Validation set
VTE: Venous thromboembolism
WBC: White blood cell
