# Supplementary material for: Venous thromboembolism in COVID-19 patients and prediction model: a multicenter cohort study
Source: BMC Infect Dis. 2022 May 13;22:462. doi: 10.1186/s12879-022-07421-3 (PMC9100286; doi:10.1186/s12879-022-07421-3)
Supplement: Supplementary file 1 — Additional file 1. Supplemental materials of COVID-19 venous thromboembolism prediction model. [file 12879_2022_7421_MOESM1_ESM.docx]

**SUPPLEMENTARY MATERIALS**

**Venous Thromboembolism in COVID-19 Patients and Prediction Model: A Multicenter Cohort Study**

Yi Lee, Qasim Jehangir, Pin Li, Deepthi Gudimella, Pooja Mahale, Chun-Hui Lin, Dinesh R. Apala, Geetha Krishnamoorthy, Abdul R. Halabi, Kiritkumar Patel, Laila Poisson, Venugopal Balijepally, Anupam A. Sule, Girish B. Nair

**Table of Contents**

Page 2 – 5: Table S1: Dictionary of variables in Southeastern Michigan Consortium
 Registry Database

Page 6: Table S2: ICD-10 and Consortium Sheet codes for in-hospital venous
 thromboembolic events, including acute deep vein thrombosis and acute
 pulmonary embolism

Page 7 – 12: Table S3: Univarate Analysis for predictors of acute venous thromboembolic events

Page 13: Table S4: Functions and Packages Used in the analysis

Page 14 – 15: Table S5A: Correlation of variables

Page 16: Table S5B: Predictors that were excluded

Page 17 – 18: Figure S1A: Scree plot of principal components analysis

Page 19 Figure S1B: Biplot of the Principal Components Analysis

Page 20: Figure S2: Decision tree of VTE model in COVID-19 patients

**Supplementary Table 1. Dictionary of variables in Southeastern Michigan Consortium Registry Database.**

| **Category** | **Predictor variables** | **Description** | **Values** |
| --- | --- | --- | --- |
| Personal Information | Patient Study ID | Unique alphanumeric value for each patient |  |
|  | AdmissionNumber | Determines if the patient is admitted the first time in the hospital or is a revisitor | 1 = New Patient 2 = Revisitor 3 = Revisitor |
|  | Gender | Gender of the patient | Male=0, Female=1 |
|  | Race/ethnicity | Race/ethnicity of the patient | 1 = American Indian or Alaskan Native 2 = Asian or Pacific Islander 3 = Black 4 = White 5 = Hispanic  6 = Others 7 = Unknown |
|  | AgeAtFirstAdmission | Age of the patient at first admission | Number>0 |
|  | BMICat | Body mass index of the patient | <18.5 = 1 18.5-24.9 = 2  25-29 = 3  >30 = 4 |
| Hospital Summary | HadCOVIDDx | Was a COVID-19 diagnosis made/treated in this inpatient episode? | 1=yes, 0=no |
|  | HospitalLOS | Days from ED to Discharge | Number>0 |
|  | TotalICULOS | Days spent in the intensive care unit | Number>0 |
|  | CovidVentDays | The number of days on mechanical ventilation | Number>=0 |
|  | CPR | Was cardiopulmonary resuscitation performed? | 1=yes, 0=no |
|  | PresentingO2Sat | Patients’ oxygen saturation on admission | Numeric, 0-100 |
|  | O2device | Patients require oxygen support on admission | Nasal cannula/Non-rebreather mask Ventilator High-flow nasal cannula None = breathing on room air Other |
|  | SOFAScore | Sequential Organ Failure Assessment (SOFA) score at the time of presentation to the hospital for this episode of care |  |
| Lab Values | WBC | White blood cell count of the patient | Numeric>0 |
|  | Neutrophil-lymphocyte ratio | Neutrophil-lymphocyte ratio of the patient | Numeric>0 |
|  | BNP | B-type natriuretic peptide content of the patient | Numeric>0 |
|  | CRP | C-reactive protein content of the patient | Numeric>0 |
|  | D-dimer | D-dimer content of the patient | Numeric>0 |
|  | Ferritin | Ferritin content of the patient | Numeric>0 |
|  | LDH | Lactate dehydrogenase content of the patient | Numeric>0 |
|  | ALT | Alanine transaminase content of the patient | Numeric>0 |
|  | AST | Aspartate aminotransferase content of the patient | Numeric>0 |
|  | BUN | Blood urea nitrogen content of the patient | Numeric>0 |
|  | Creatinine | Creatinine content of the patient | Numeric>0 |
|  | Potassium | Potassium content of the patient | Numeric>0 |
|  | T.bilirubin | Total bilirubin content of the patient | Numeric>0 |
|  | PlateletCount | Platelet count of the patient | Numeric>0 |
| Past medical history of the patient | Surgery | If patient had any previous surgeries | Coronary artery bypass graft Cardiac stent Lung transplant Pulmory lobectomy No = had no surgery |
|  | AtrialFibrillation | Atrial fibrillation, a type of cardiac medical history | 1=yes, 0=no |
|  | CAD | Coronary Artery Disease, a type of cardiac medical history | 1=yes, 0=no |
|  | HeartFailure | Heart failure, a type of cardiac medical history | 1=yes, 0=no |
|  | Hyperlipidemia | A type of cardiac medical history | 1=yes, 0=no |
|  | Hypertension | A type of cardiac medical history | 1=yes, 0=no |
|  | MI | Myocardial Infraction, a type of cardiac medical history | 1=yes, 0=no |
|  | PriorPercutaneousCororyInterventions | Prior cardiac catheterization, a type of cardiac medical history | 1=yes, 0=no |
|  | ValvularHeartDisease | Valvular heart disease, a type of cardiac medical history | 1=yes, 0=no |
|  | VentricularTachycardia | Ventricular tachycardia, a type of cardiac medical history | 1=yes, 0=no |
|  | Diabetes | A type of medical history | 1=yes, 0=no |
|  | Cancer | Other medical history | 1=yes, 0=no |
|  | Autoimmune | Other medical history | 1=yes, 0=no |
|  | COPD | Pulmonary medical history | 1=yes, 0=no |
|  | InterstitialLungDisease | Interstitial lung disase, a type of pulmonary medical history | 1=yes, 0=no |
|  | PulmoryFibrosis | Pulmonary fibrosis, a type of pulmonary medical history | 1=yes, 0=no |
|  | PulmoryHypertension | Pulmonary hypertension, a type of pulmonary medical history | 1=yes, 0=no |
|  | VenousThromboembolism | Priot medical history of venous thromboembolism | 1=yes, 0=no |
|  | CKD | Chronic kidney disease, a type of renal medical history | 1=yes, 0=no |
|  | Thyroid_merge | Thryoid disease, a type of endocrine medical history | 1=yes, 0=no |
|  | Leukemia | Hematological history | 1=yes, 0=no |
|  | SickleCellDisease | Sickle cell disease, a type of hematological history | 1=yes, 0=no |
|  | Cirrhosis | Liver medical history | 1=yes, 0=no |
|  | HepatocellularCarcimoma | Hepatocellular carcinoma, a type of liver medical history | 1=yes, 0=no |
|  | MultipleSclerosis | Neurological medical history | 1=yes, 0=no |
|  | TIA.Stroke | Cerebrovascular accident including transient ischemic attack and stroke, a type of neurological medical history | 1=yes, 0=no |

This dictionary shows the categories of variables of (1). Personal history, (2). Hospital summary with presenting vitals, oxygen devices, length of stay in intensive care unit, etc (3). Laboratroy values, (4). Past medical history.

**Supplementary Table 2. International Classification of Diseases–Tenth Revision and Consortium Sheet codes for in-hospital venous thromboembolic events, including acute deep vein thrombosis and acute pulmonary embolism.**

|  | **International Classification of Diseases–Tenth Revision & Consortium Sheet Codes** |
| --- | --- |
| **Acute pulmonary embolism** | Discharge Diagnosis: Acute Pulmonary embolism  *I26.0 I26.01 I26.02 I26.09 I26.9 I26.90 I26.92 I26.93 I26.94 I26.99* |
| **Other acute venous thrombosis and embolism** | Discharge Diagnosis: Acute venous thrombosis and embolism  *I81 I82.2 I82.21  I82.210 I82.22 I82.220 I82.29 I82.290 I82.4 I82.40I82.401  I82.402 I82.403 I82.409 I82.41 I82.411 I82.412  I82.413 I82.419 I82.42 I82.421 I82.422 I82.423 I82.429  I82.43 I82.431 I82.432 I82.433 I82.439 I82.44 I82.441 I82.442 I82.443 I82.449 I82.45 I82.451 I82.452  I82.453 I82.459 I82.46 I82.461 I82.462 I82.463 I82.469 I82.49 I82.491 I82.492 I82.493 I82.499 I82.4Y I82.4Y1 I82.4Y2  I82.4Y3 I82.4Y9 I82.4Z I82.4Z1 I82.4Z2 I82.4Z3 I82.4Z9  I82.6  I82.60 I82.601 I82.602  I82.603 I82.609 I82.61 I82.611 I82.612 I82.613 I82.619  I82.62 I82.621  I82.622  I82.623  I82.629 I82.A  I82.A1 I82.A11 I82.A12 I82.A13 I82.A19  I82.C1 I82.C11 I82.C12 I82.C13 I82.C19  I82.8 I82.81 I82.811 I82.812  I82.813 I82.819 I82.89 I82.890  I82.9  I82.90* |

**Supplementary Table 3. Univarate Analysis for predictors acute venous thromboembolic events.**

|  | **Variable** | | **Odds Ratio^¥^ (95% CI, p value)** |
| --- | --- | --- | --- |
|  | **Gender** | Male | 0.98 (0.77-1.25, p=0.865) |
|  | **Race/ethnicity** | American Indian or Alaskan Native | - |
|  |  | Asian or Pacific Islander | 0.11 (0.00-3.05, p=0.137) |
|  |  | Black | 0.56 (0.09-10.57, p=0.590) |
|  |  | White | 0.51 (0.09-9.60, p=0.530) |
|  |  | Hispanic | 0.11 (0.00-3.05, p=0.137) |
|  |  | Others | 0.26 (0.03-5.52, p=0.261) |
|  |  | Unknown | 0.64 (0.09-12.85, p=0.697) |
|  | Age (years) | [19.0,101.0] | 1.00 (1.00-1.01, p=0.242) |
|  | Body mass index (kg/m^2^), mean | <18.5 | - |
|  |  | 18.5-24.9 | 0.98 (0.46-2.43, p=0.955) |
|  |  | 25-29 | 0.75 (0.35-1.84, p=0.482) |
|  |  | >30 | 0.72 (0.35-1.77, p=0.430) |
|  | Readmission | 1 | 0.32 (0.19-0.58, p<0.001) * |
|  | Hospital length of stay (days) | [1.0,51.0] | 1.06 (1.05-1.07, p<0.001)* |
|  | Total intensive care unit length of stay (days) | [0.0,46.0] | 1.05 (1.04-1.07, p<0.001)* |
|  | Mechanical ventilation (days) | [0.0,45.0] | 1.06 (1.04-1.08, p<0.001)* |
|  | Cardiopulmonary resuscitation | | 0.95 (0.69-1.35, p=0.778) |
|  | Oxygen device | None | - |
|  |  | Nasal cannula/non-rebreather mask | 1.48 (1.10-1.96, p=0.008)* |
|  |  | Ventilator | 3.14 (1.62-5.66, p<0.001)* |
|  |  | Other | 2.54 (0.95-5.74, p=0.038)* |
|  |  | High-flow nasal cannula | 12.59 (4.94-31.64, p<0.001)* |
| Vitals on presentation | Oxygen Saturation (%) | [2.0,100.0] | 0.99 (0.97-1.01, p=0.259) |
|  | Heart rate (beats/minute) | [11.0,209.0] | 1.01 (1.01-1.02, p<0.001)* |
|  | Respiratory rate (breaths/minute) | [1.0,135.0] | 1.01 (0.99-1.03, p=0.208) |
|  | Diastolic blood Pressure (mmHg) | [0.0,235.0] | 1.00 (1.00-1.01, p=0.370) |
|  | Systolic blood pressure (mmHg) | [45.0,266.0] | 1.00 (0.99-1.00, p=0.164) |
| Labs on presentation | White blood cell count (K/uL) | [0.2,85.9] | 1.06 (1.04-1.08, p<0.001)* |
|  | Lymphocytes (K/uL) | [0.0,89.0] | 0.98 (0.97-1.00, p=0.012)* |
|  | Neutrophils (K/uL) | [0.0,98.0] | 1.00 (0.99-1.00, p=0.761) |
|  | Hemoglobin A1c (%) | [4.5,17.1] | 0.89 (0.77-1.02, p=0.114) |
|  | B-type natriuretic peptide (pg/mL) | [5.0,3924.0] | 1.00 (1.00-1.00, p=0.957) |
|  | Ferritin (ng/mL) | [4.0,48174.0] | 1.00 (1.00-1.00, p=0.688) |
|  | Fibrinogen (mg/dL) | [98.0,1155.0] | 1.00 (1.00-1.00, p=0.212) |
|  | C-reactive protein (mg/dL) | [0.1,47.6] | 1.04 (1.02-1.05, p<0.001)* |
|  | D-dimer (μg/mL) | [0.2,26.8] | 1.19 (1.15-1.23, p<0.001)* |
|  | Interleukin-6 (pg/mL) | [2.5,918.5] | 1.00 (1.00-1.00, p=0.002)* |
|  | Lactate dehydrogenase (U/L) | [69.0,14007.0] | 1.00 (1.00-1.00, p=0.062) |
|  | Alanine transaminase (U/L) | [3.0,8518.0] | 1.00 (1.00-1.00, p=0.537) |
|  | Aspartate aminotransferase (U/L) | [7.0,9251.0] | 1.00 (1.00-1.00, p=0.025)* |
|  | Procalcitonin (ng/mL) | [0.1,100.0] | 0.99 (0.96-1.01, p=0.514) |
|  | Blood urea nitrogen (mg/dL) | [3.0,241.0] | 1.01 (1.00-1.01, p=0.003)* |
|  | Creatinine (mg/dL) | [0.2,21.5] | 1.03 (0.96-1.08, p=0.394) |
|  | Potassium (meq/L) | [1.3,9.5] | 1.28 (1.08-1.51, p=0.003)* |
|  | Total bilirubin (mg/dL) | [0.1,37.7] | 1.12 (1.03-1.23, p=0.007)* |
|  | Hemoglobin (gm/dL) | [6.4,16.0] | 1.28 (0.81-2.26, p=0.311) |
|  | Lactate (mmol/L) | [0.4,29.1] | 1.02 (0.90-1.12, p=0.714) |
|  | Platelet Count (K/uL) | [18.0,1115.0] | 1.00 (1.00-1.00, p<0.001)* |
| Social history |  |  | - |
|  | Smoker | | 0.94 (0.57-1.47, p=0.789) |
|  | Alcohol Use | | 1.55 (0.64-3.21, p=0.284) |
|  | Marijuana Use | | 1.88 (0.44-5.55, p=0.314) |

^¥^Odds ratio was calculated as [Probability of VTE, given Variable/Probability of VTE given not Variable)]/[Probability of not VTE given variable)/Probability of not VTE given not Variable)]

**Supplementary Table 3. (Continued)**

|  | **Variable** | | **Odds Ratio (95% CI, p value)** |
| --- | --- | --- | --- |
| In-hospital medications      Home medications | Inpatient anticoagulation therapeutic dose | | 5.59 (4.27-7.32, p<0.001) * |
|  | Inpatient anticoagulation prophylactic dose | | 0.25 (0.19-0.35, p<0.001) * |
|  | Non-steroidal anti-inflammatory drugs | | 1.85 (0.59-4.88, p=0.242) |
|  | Azithromycin | | 2.10 (0.58-6.07, p=0.203) |
|  | Hydroxychloroquine | | 2.09 (0.11-13.54, p=0.509) |
|  | Angiotensin-converting enzyme inhibitors | | 1.36 (0.57-3.03, p=0.472) |
|  | Angiotensin receptor blockers | | 1.17 (0.38-2.99, p=0.766) |
|  | Beta blockers | | 0.55 (0.21-1.27, p=0.183) |
|  | Diuretics | | 0.94 (0.40-2.09, p=0.887) |
|  | Statins | | 0.56 (0.24-1.24, p=0.169) |
|  | Warfarin | | 1.62 (0.24-6.27, p=0.540) |
|  | Aspirin | | 0.64 (0.24-1.47, p=0.316) |
|  | P2Y12 inhibitors | | 1.76 (0.27-6.90, p=0.474) |
|  | Direct oral anticoagulants | | NA |
|  | Other anticoagulants | | NA |
|  | Corticosteroids | | 1.48 (0.41-4.15, p=0.495) |
|  | Proton pump inhibitors | | 1.75 (0.77-3.81, p=0.169) |
| Lab values (Maximum and Minimum) | White blood cell count Max |  | 1.03 (1.02-1.05, p<0.001)* |
|  | Lymphocytes Min |  | 0.97 (0.95-0.99, p=0.001)* |
|  | Neutrophils Max |  | 1.03 (1.01-1.04, p=0.001)* |
|  | Hemoglobin A1c Min |  | 0.90 (0.77-1.02, p=0.118) |
|  | B-type natriuretic peptide Max |  | 1.00 (1.00-1.00, p=0.928) |
|  | Ferritin Max |  | 1.00 (1.00-1.00, p=0.734) |
|  | Lactate Max |  | 1.08 (0.99-1.17, p=0.073) |
|  | C-reactive protein Max |  | 1.04 (1.03-1.06, p<0.001)* |
|  | D-dimer Max |  | 1.21 (1.16-1.26, p<0.001)* |
|  | Interleukin-6 Max |  | 1.00 (1.00-1.00, p=0.002)* |

**Supplementary Table 3. (Continued)**

|  | **Variable** | **Odds Ratio (95% CI, p value)** |
| --- | --- | --- |
| **Prior Medical History** | Atrial fibrillation | 0.66 (0.40-1.03, p=0.086) |
|  | Coronary artery disease | 0.74 (0.51-1.03, p=0.088) |
|  | Deep vein thrombosis | 2.67 (1.81-3.86, p<0.001)* |
|  | Heart failure | 0.80 (0.53-1.17, p=0.267) |
|  | Hyperlipidemia | 0.85 (0.65-1.10, p=0.212) |
|  | Hypertension | 0.85 (0.63-1.17, p=0.312) |
|  | Myocardial infarction | 1.21 (0.79-1.79, p=0.363) |
|  | Valvular heart disease | 1.08 (0.59-1.83, p=0.790) |
|  | Ventricular tachycardia | 0.85 (0.05-4.29, p=0.876) |
|  | Diabetes mellitus | 0.90 (0.69-1.17, p=0.446) |
|  | Malignancy | 1.24 (0.91-1.68, p=0.169) |
|  | Autoimmune disease | 1.24 (0.69-2.08, p=0.432) |
|  | Chronic obstructive pulmonary disease | 0.65 (0.43-0.95, p=0.034)* |
|  | Interstitial lung disease | 1.38 (0.07-7.59, p=0.760) |
|  | Pulmonary embolism | 3.40 (2.15-5.21, p<0.001)* |
|  | Pulmonary fibrosis | 0.79 (0.04-3.95, p=0.819) |
|  | Pulmonary hypertension | 0.70 (0.17-1.93, p=0.555) |
|  | Other venous thromboembolism | 3.34 (2.39-4.61, p<0.001)* |
|  | Chronic kidney disease | 1.26 (0.86-1.80, p=0.222) |
|  | End-stage renal disease | 1.16 (0.51-2.29, p=0.694) |
|  | Thyroid disease | 1.01 (0.69-1.44, p=0.948) |
|  | Sickle cell disease | 1.01 (0.05-5.20, p=0.996) |
|  | Ascites | 8.38 (1.64-38.24, p=0.006)* |
|  | Liver cirrhosis | 1.01 (0.16-3.44, p=0.994) |
|  | Hepatocellular carcinoma | 3.70 (0.18-29.00, p=0.258) |
|  | Non-alcoholic steatosis hepatitis | 3.70 (0.18-29.00, p=0.258) |
|  | Multiple sclerosis | 0.61 (0.03-2.99, p=0.634) |
|  | Transient ischemic attack or stroke | 0.81 (0.53-1.20, p=0.314) |

**Supplemenatry Table 3 (Continued).** Univariate Analysis for venous thromboembolic events including baseline characteristics, initial vital signs on presentation, laboratory on presentation, social history, medication history prior to admission, and past medical history, anticoagulation during hospitalization. *p<0.05. Abbreviations: Max, maximum; min, minimum.

**Supplemenatry Table 4. Functions and Packages Used in the analysis.**

| ***JMP Pro functions***  ## Correlations for continuous variables:  Analyze > Multivariate Methods > Multivariate.  Select Nonparametric Correlation  Select Spearman’s Rho (r)  ## Random sampling:  Tables > Subset, random split ratio: 30:70  ## LR, MLR model:  Analyze > Fit Model  ## Decision trees and random forest:  Analyze > Predictive Modeling > Partition  ***UVA and PCA R packages***  ##library(finalfit)  forfit1 = forfit %>% filter(Event == 'Venous' \| Event == 'No Event')   ## summary table 1  tab1 = forfit1 %>%    summary_factorlist()  ## univariate analysis  uva1 = forfit1 %>%    finalfit()  ##pca  library(factoextra)  cov.pca2.res <- prcomp(cov.pca2, scale = TRUE)  fviz_eig(cov.pca2.res) #scree plot  fviz_pca_biplot(cov.pca2.res, repel = TRUE, col.var = "navyblue", col.ind = "grey70") #biplot |
| --- |

**Supplementary Table 5A. Correlation of variables.**

| **Variable 1** | **Variable 2** | **Spearman's rho** | **Significance Value** |
| --- | --- | --- | --- |
| AST | ALT | 0.7 | <0.0001 |
| Creatinine | BUN | 0.74 | <0.0001 |
| BNPMax | BNP | 0.9765 | <0.0001 |
| CRPMax | CRP | 0.6695 | <0.0001 |
| FerritinMax | Ferritin | 0.9061 | <0.0001 |
| D-dimerMax | D-dimer | 0.8465 | <0.0001 |
| LDHMax | LDH | 0.8394 | <0.0001 |
| WBCMax | WBC | 0.5576 | <0.001 |
| HadCOVIDDx | AdmissionNumber | -0.8551 | <0.0001 |
| CovidVentDays | TotalICUDays | 0.7819 | <0.0001 |
| Neutrophil-lymphocyte ratio Max | LymphocytesMin | -0.9791 | <0.0001 |
| Neutrophil-lymphocyte ratio Max | NeutrophilsMax | 0.7606 | <0.0001 |
| Neutrophil-lymphocyte ratio Max | Lymphocytes | -0.6516 | <0.0001 |
| Neutrophil-lymphocyte ratio | Lymphocytes | -0.7956 | <0.0001 |
| Neutrophil-lymphocyte ratio | Neutrophils | 0.96 | <0.0001 |
| Neutrophil-lymphocyte ratio | Lymphocytes | -0.7956 | <0.0001 |
| Neutrophil | Lymphocytes | -0.7281 | <0.0001 |
| NeutrophilsMax | LymphocytesMin | -0.6639 | <0.0001 |
| LymphocytesMin | Lymphocytes | 0.635 | <0.0001 |
| VenousThromboembolism | DeepVeinThrombosis | 0.7952 | <0.0001 |
| VenousThromboembolism | PulmoryEmbolism | 0.6593 | <0.0001 |
| SystolicBP | DiastolicBP | 0.5585 | <0.0001 |
| ip_AC_prophylatic | ip_AC_therapeutic | -0.7434 | <0.0001 |

23 groups of variables that are highly correlated based on Spearman’s coefficient. The variables are explained in the dictionary of variables in Supplementary Table 1.

**Supplementary Table 5B.** **Predictors that were excluded.**

| **Predictor** |
| --- |
| BNPMax |
| CRPMax |
| FerritinMax |
| D-dimerMax |
| LDHMax |
| WBCMax |
| TotalICUDays |
| LymphocytesMin |
| NeutrophilsMax |
| N/L ratio Max |
| Neutrophils |
| Lymphocytes |
| DeepVeinThrombosis |
| PulmoryEmbolism |

The variables are explained in Supplementary Table 1.


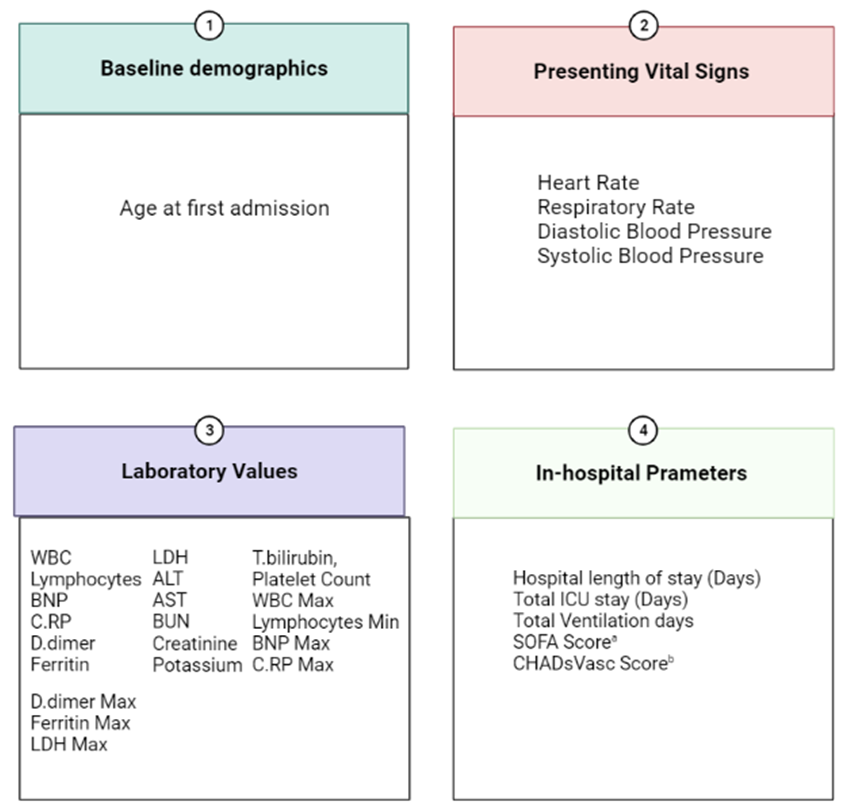


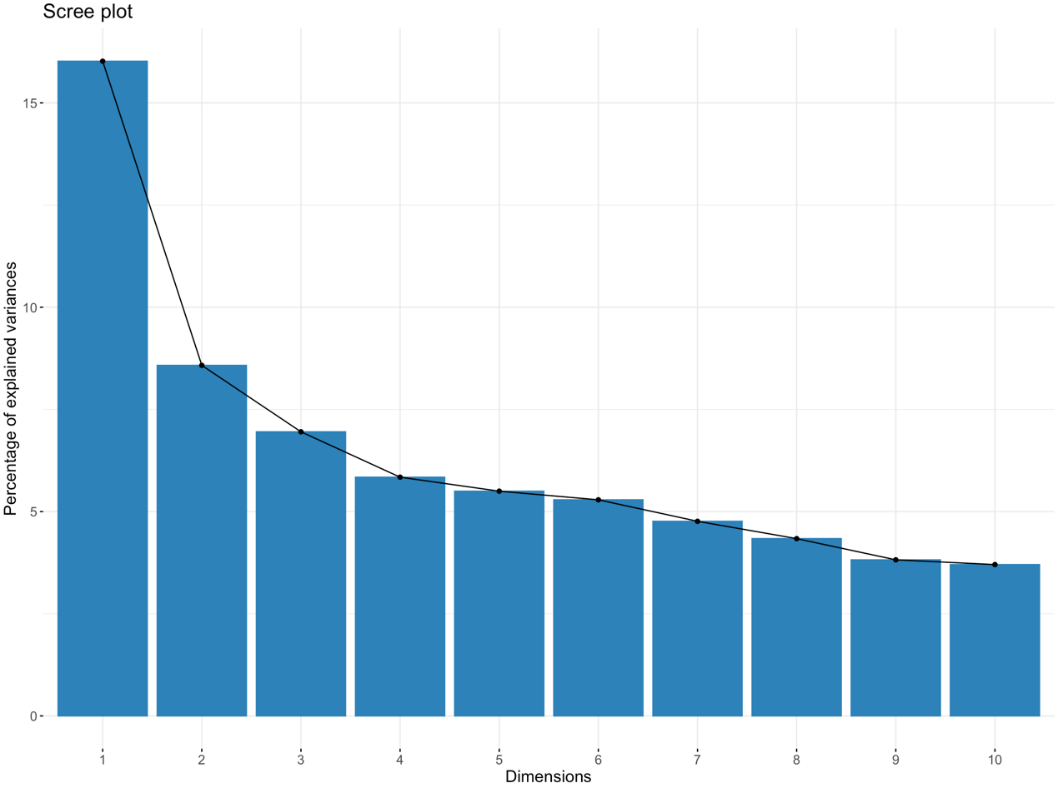


**Supplementary Figure 1A.** **Scree plot of principal components analysis (PCA).** Patient without missing data (N=1443) from the cohort were included in the PCA. 32 continuous variables were included in PCA. In the scree plot, the 1^st^ component explains only about 16% variations of the data and 24.6% of the variations can be explained by the first 2 components. The variables are explained in Supplementary Table 1.


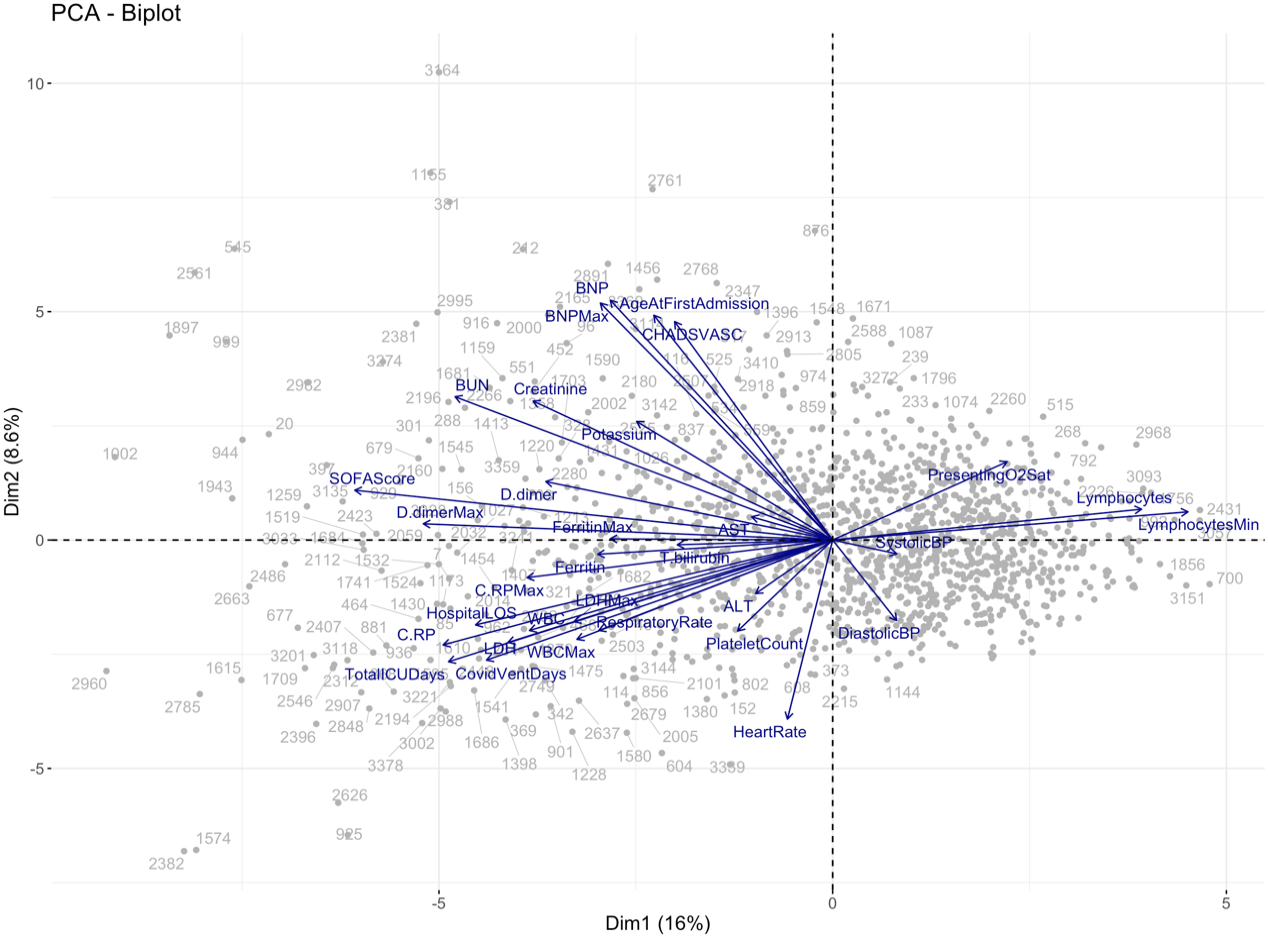


**Supplemenatry Figure 1B. Biplot of the Principal Components Analysis.** The top 2 components are the x and y axis and the k vectors (blue arrows) show how strongly each characteristic influence a principal component. PC1 explained 16% of the total variation and PC2 explained 8.6%. Most of the vectors are horizontal, and they strongly influence PC1. Many of them are positive correlated as they are close to each other. Among which, presenting heart rate (HR) and blood pressure (BP) strongly influence PC2. Other variables are explained in Supplementary Table 1.

**Supplemenatry Figure 2.** **Decision tree of VTE model in COVID-19 patients.** This diagram delienated the nodes of our decision tree model. The first split was on the node of therapeutic anticoagulation in hospitalized patients into yes or no. Each one of this splits further on BUN, hospital length of stay, D-dimer and history of VTE.

Abbreviations: BUN, blood urea nitrogen; VTE, venous thromboembolism
